# OGA heterozygosity suppresses intestinal tumorigenesis in *Apc*^min/+^ mice

**DOI:** 10.1038/oncsis.2014.24

**Published:** 2014-07-07

**Authors:** Y R Yang, H-J Jang, S Yoon, Y H Lee, D Nam, I S Kim, H Lee, H Kim, J H Choi, B H Kang, S H Ryu, P-G Suh

**Affiliations:** 1 School of Life Sciences, Ulsan National Institute of Science and Technology, Ulsan, Republic of Korea; 2 Division of Molecular and Life Science, Pohang University of Science and Technology, Pohang, Kyungbuk; 3 Research Institute, Graduate School of Cancer Science and Policy, National Cancer Center, Goyang, Republic of Korea; 4 Department of Pathology and Yonsei University College of Medicine, Seoul, Republic of Korea

## Abstract

Emerging evidence suggests that aberrant O-GlcNAcylation is associated with tumorigenesis. Many oncogenic factors are O-GlcNAcylated, which modulates their functions. However, it remains unclear how O-GlcNAcylation and O-GlcNAc cycling enzymes, O-GlcNAc transferase (OGT) and O-GlcNAcase (OGA), affect the development of cancer in animal models. In this study, we show that reduced level of OGA attenuates colorectal tumorigenesis induced by Adenomatous polyposis coli (*Apc*) mutation. The levels of O-GlcNAcylation and O-GlcNAc cycling enzymes were simultaneously upregulated in intestinal adenomas from mice, and in human patients. In two independent microarray data sets, the expression of OGA and OGT was significantly associated with poor cancer-specific survival of colorectal cancer (CRC) patients. In addition, OGA heterozygosity, which results in increased levels of O-GlcNAcylation, attenuated intestinal tumor formation in the *Apc*^min/+^ background. *Apc*^min/+^ *OGA*^+/−^ mice exhibited a significantly increased survival rate compared with *Apc*^min/+^ mice. Consistent with this, *Apc*^min/+^ *OGA*^+/−^ mice expressed lower levels of Wnt target genes than *Apc*^min/+^. However, the knockout of OGA did not affect Wnt/β-catenin signaling. Overall, these findings suggest that OGA is crucial for tumor growth in CRC independently of Wnt/β-catenin signaling.

## Introduction

O-GlcNAc transferase (OGT) and O-GlcNAcase (OGA) are enzymes that regulate the addition and removal of monosaccharides of O-linked β-*N*-acetylglucosamine to the Ser and Thr residues (O-GlcNAc) of target proteins, respectively.^1^ Increasing evidence suggests that OGT and/or OGA expression and O-GlcNAc levels are altered in different types of cancer, including colon, lung,^2^ breast,^3, 4^ liver,^5^ prostate,^6, 7^ pancreas^8^ and bladder.^9^ Furthermore, tumor aggressiveness is closely associated with the levels of O-GlcNAcylation, OGT and/or OGA. Consistent with this, various O-GlcNAc-modified proteins are perturbed in tumorigenesis. For example, O-GlcNAc-modified proteins are involved in cancer-relevant processes such as transcriptional regulation, cell proliferation and the cell cycle.^10^ The activity of many oncogenic factors, including c-Myc,^11^ β-catenin,^12^ p53^13^ and FoxM1,^14^ is regulated by direct O-GlcNAcylation. In addition, O-GlcNAc cycling is required for the precise control of the cell cycle, suggesting an essential role for O-GlcNAc cycling enzymes in cell proliferation.^12, 15, 16^

Colorectal cancer (CRC) is the third common cancer worldwide. Adenomatous polyposis coli (*Apc*) is a tumor suppressor gene that is mutated in ∼80% of sporadic adenomatous polyps and CRCs.^17^ Mutant APC causes the oncogenic activation of β-catenin. Notably, β-catenin is directly O-GlcNAcylated. Several studies have suggested that the O-GlcNAcylation of β-catenin affects its transcriptional activity and subcellular localization.^12, 18^ Moreover, higher levels of O-GlcNAcylation and OGT expression are found in colon tumors than in corresponding non-tumorous mucosal tissues.^2^ Although current evidence implicates O-GlcNAcylation in CRC, it remains unclear exactly how the levels of O-GlcNAcylation or O-GlcNAc cycling enzymes affect colorectal tumorigenesis. To evaluate the role of OGA in colorectal tumorigenesis, we investigated whether OGA heterozygosity could alter CRC susceptibility in *Apc*^min/+^ mice.

We found that the levels of O-GlcNAcylation and O-GlcNAc cycling enzymes were elevated simultaneously in human and mouse colorectal tumors. Increased expression of the O-GlcNAc enzymes OGT and OGA was correlated significantly with poor survival. Therefore, OGA heterozygosity attenuates colorectal tumorigenesis in *Apc*^min/+^ mice. Although β-catenin is O-GlcNAcylated, elevated levels of O-GlcNAcylation did not affect Wnt/β-catenin signaling. Taken together, our observation supports the hypothesis that OGA plays a key role in intestinal tumorigenesis.

## Results and discussion

### Increased O-GlcNAcylation and O-GlcNAc cycling enzymes in colorectal adenomas

**Figure 1:**
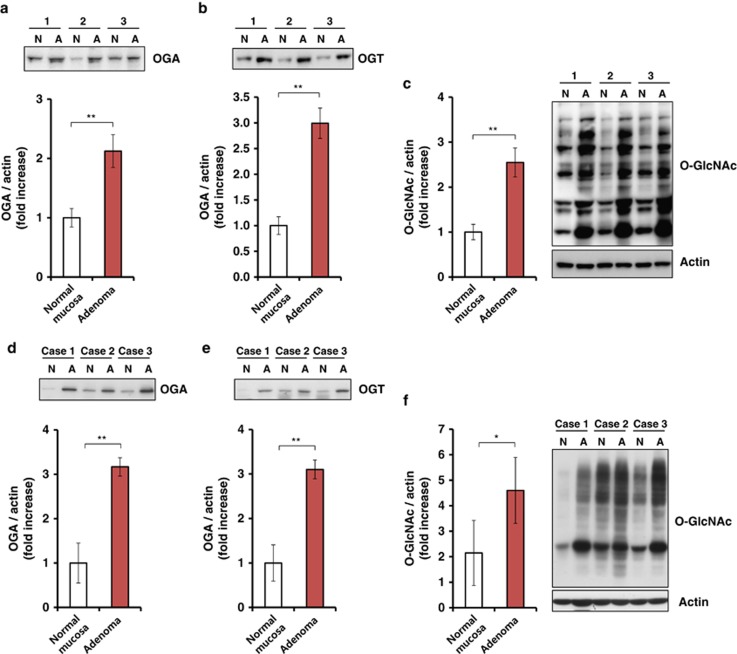
The levels of O-GlcNAc cycling enzymes are elevated in mice and human patients with colonic adenomas. (**a–c**) The levels of OGA (**a**), OGT (**b**) and O-GlcNAc (**c**) in normal mucosa (N) and *Apc*^min/+^ mouse adenomas (A) were compared by western blotting using the following antibodies: the anti-OGT and -OGA polyclonal antibodies, which had been generated previously and were used as described,^25^ O-GlcNAc (RL2) (MA1-072; Thermo Fisher Scientific Inc., Waltham, MA, USA) and anti-β-actin (691001; MP Biomedicals, Santa Ana, CA, USA). Densitometry was performed to quantify the immunoblots, and the ratios of OGA, OGT and O-GlcNAc to β-actin were determined. (**d–f**) The same experiment was performed with CRC samples isolated from human patients. Densitometry was performed on immunoblots as described above. Error bars represent the standard deviation (s.d.; *n*=3). **P<0.005, *P<0.05 (Student's *t*-test). The human specimens were obtained from the Department of Pathology, Yonsei University (Seoul, Korea), and from the Liver Cancer Specimen Bank of the National Research Resource Bank Program of the Korea Science and Engineering Foundation of the Ministry of Science and Technology. Authorization for the use of these tissues for research purposes was obtained from the Institutional Review Board of Yonsei University of College of Medicine (IRB number: 4-2012-0026).

Previous studies have suggested that abnormal levels of O-GlcNAcylation and O-GlcNAc enzymes are closely linked to tumorigenesis. However, it is unclear whether O-GlcNAcylation plays a role in colorectal tumorigenesis *in vivo*. First, we analyzed the expression pattern of O-GlcNAcylation and O-GlcNAc cycling enzymes in CRC tissues from *Apc*^min/+^ mice and human patients. Colonic homogenates of normal mucosa and adenomas from *Apc*^min/+^ mice were analyzed by immunoblotting to assess the levels of O-GlcNAcylation and O-GlcNAc cycling enzymes. Interestingly, we observed that O-GlcNAcylation was increased significantly (greater than twofold) in colonic adenomas compared with normal mucosa (Figures 1a and b). Colonic adenomas also expressed higher levels of OGT and OGA than normal mucosa (Figures 1c and d). Consistent findings were observed in human patients with CRC (Figures 1e–h). Surprisingly, both OGT and OGA enzymes were increased simultaneously in colonic adenomas. This suggests that the upregulation of OGA compensates for the increased O-GlcNAcylation. Conversely, we also observed an OGA deletion, which elevates O-GlcNAcylation and downregulates OGT.^15^ These results suggest that the abnormally elevated levels of O-GlcNAcylation and its cycling enzymes are relevant to colorectal tumorigenesis.

### Association of O-GlcNAc cycling enzyme expression with survival and cancer recurrence in patients with CRC

**Figure 2:**
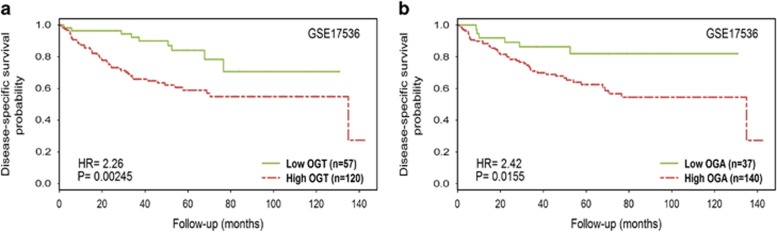
Association of the expression levels of O-GlcNac cycling enzymes with CRC patient survival and relapse. (**a**) The MCC cohort was divided into two groups according to O-GlcNac enzyme levels, and the Kaplan–Meier curves of disease-specific survival were compared. (**b**) The Norwegian patient cohort was divided into two groups according to O-GlcNac enzyme levels, and the Kaplan–Meier curves of relapse-free survival were compared. The split with the lowest *P*-value in the log-rank test was selected from among the median 60% of samples sorted by gene expression levels. The hazard ratios and *P*-values shown in the plots were calculated from Cox's proportional-hazard regression model.

Because the aberrant expression of OGT and OGA has been reported in different types of cancer, we assessed whether OGT and OGA expression exhibits prognostic value in CRC. Two independent cohorts of CRC patients from the Moffitt Cancer Center (GSE17536)^19^ and a Norwegian hospital (Norwegian; GSE30378)^20^ were analyzed for the association between OGT and OGA expression and patient survival and cancer recurrence. The Moffitt Cancer Center data set reported the disease-specific survival times for 177 CRC patients. The gene expression levels of *OGT* and *OGA* had a strong positive correlation (Pearson's correlation=0.71; *P*-value <2.2e−16), supporting the upregulation of these genes in cancer (Figures 1a and e). Expression was also significantly associated with the disease-specific survival probability: OGT and OGA had hazard ratios of 2.26 (*P*=0.00245) and 2.42 (*P*=0.0155). Patients with reduced levels of OGT and OGA exhibited improved survival rates (Figures 2a and b).

### OGA heterozygosity reduces the number of intestinal tumors and increases the survival of *Apc*^min/+^ mice

**Figure 3:**
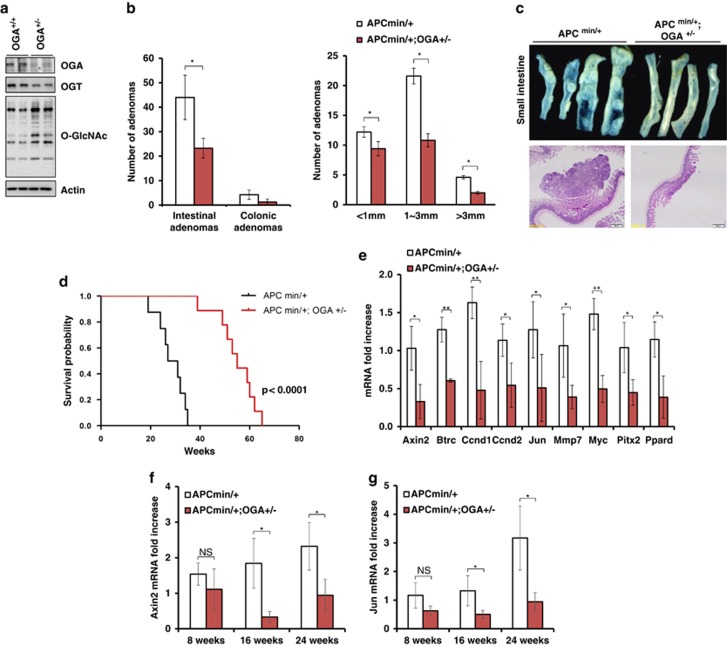
OGA heterozygosity attenuates APC-mediated intestinal tumorigenesis. (**a**) The expression of OGA was confirmed in *OGA*^+/−^ intestines. O-GlcNAc levels were elevated, and OGT was downregulated in *OGA*^+/−^ intestines. (**b**) The number (left) and size (right) of intestinal tumors. (**c**) Gross appearance of the small intestine (upper), and hematoxylin and eosin staining (lower) of intestinal adenoma sections from *Apc*^min/+^ and *Apc*^min/+^ *OGA*^+/−^ mice at 20 weeks. *n*=8 mice per group. Error bars represent ±s.e.m. **P<0.005, *P<0.05 (Student's *t*-test). (**d**) Survival rate of *Apc*^min/+^ and *Apc*^min/+^ *OGA*^+/−^ mice (Kaplan–Meier log-rank, *P*=0.0001, *n*=24 and *n*=15, respectively). *Apc*^min/+^ mice *n*=24; *Apc*^min/+^ *OGA*^+/−^ mice *n*=15. (**e**) The mRNA levels of Wnt target genes were analyzed using RT2 Profiler PCR Arrays (Qiagen, Valencia, CA, USA). Mice were killed at 8, 16 or 20 weeks and the mRNA levels of (**f**) *Axin2* and (**g**) *Jun* were analyzed by qPCR. The primer sequences (mouse) used were: *Axin2* forward, 5′-ACTCTGGAGGCTTTCGTTTG-3′ *Axin2* reverse, 5′-TTAAGTCAGCAGGGGCTCAT-3′ *Jun* forward, 5′-CCTTCTACGACGATGCCCTC-3′ *Jun* reverse, 5′-GGTTCAAGGTCATGCTCTGTTT-3′ GAPDH forward, 5′-AGGTCGGTGTGAACGGATTTG-3′ and GAPDH reverse, 5′-TGTAGACCATGTAGTTGAGGTCA-3′. Error bars represent the s.d. (*n*=3); **P<0.005, *P<0.05 (Student's *t*-test).

We next used *OGA*^+/−^ mice to explore the effect of increased OGT and OGA expression on colorectal tumorigenesis. *Apc*^min/+^ mice were crossed with *OGA*^+/−^ mice. The intestines of *OGA*^+/−^ mice showed reduced levels of both OGA and OGT expression compared with control mice (Figure 3a). We demonstrated previously that *OGA*^−/−^ mice displayed perinatal lethality, and that *OGA*^−/−^ mouse embryonic fibroblasts downregulate OGT, which might compensate for the increased levels of O-GlcNAcylation.^15^ Therefore, *OGA*^+/−^ mice are the correct model for assessing the effect of reduced OGT and OGA expression on colorectal tumorigenesis. A quantitative analysis of tumor burden revealed a significant twofold reduction in the number and size of adenomas in the small intestine of *Apc*^min/+^ *OGA*^+/−^ compared with *Apc*^min/+^ mice (Figure 3a). *Apc*^min/+^ *OGA*^+/−^ mice displayed fewer and smaller tumors along the intestinal tract (Figures 3b and c). *Apc*^min/+^ *OGA*^+/−^ mice exhibited a significantly increased survival rate compared with *Apc*^min/+^ mice, and all *Apc*^min/+^ mice died within 8 months. In contrast, *Apc*^min/+^ *OGA*^+/−^ mice showed dramatically increased survival rates (Figure 3d).

### Decreased levels of Wnt target genes in *Apc*^min/+^*OGA*^+/−^ mice

Based on the attenuated intestine tumor formation in *Apc*^min/+^ *OGA*^+/−^ mice, we next analyzed molecular changes. We examined whether OGA heterozygosity affects the expression of known Wnt/β-catenin target genes, which are upregulated in APC-mediated intestinal tumors. Nine genes were decreased in *Apc*^min/+^ *OGA*^+/−^ intestines compared with *Apc*^min/+^ intestines (Figure 3e). We observed that the Wnt target genes *Axin2* and *Jun* were not significantly changed in 8-week-old *Apc*^min/+^ *OGA*^+/−^ mice compared with age-matched *Apc*^min/+^ controls. However, dramatically reduced expression of *Axin2* and *Jun* was observed in 16- and 24-week-old animals (Figures 3f and g). These results suggest that, although reduced OGA levels do not influence APC-mediated tumor initiation, OGA plays critical roles in tumor growth. Indeed, OGA-deficient cells exhibit a reduced growth rate.^15^ In addition, OGT knockdown attenuates the growth of breast,^14^ lung and colon cancer cells.^2^ These results suggest that reduced OGT and OGA levels disrupt the dynamic regulation of O-GlcNAcylation, which attenuates cell growth.

### OGA disruption does not affect Wnt/β-catenin signaling

**Figure 4:**
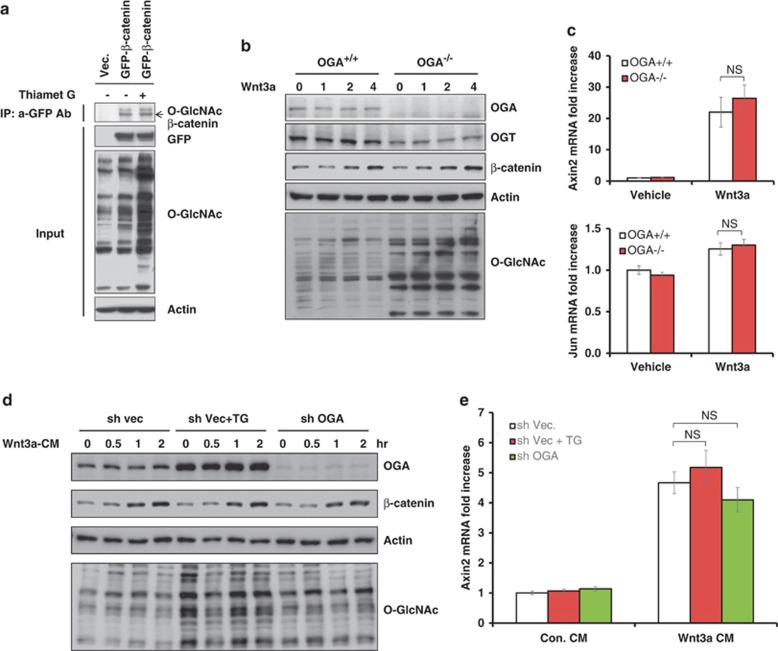
Elevated O-GlcNAcylation does not affect Wnt/β-catenin signaling. (**a**) β-Catenin is O-GlcNAcylated. HEK-293 cells were transfected with empty vector or a GFP-β-catenin expression vector. GFP-β-catenin-transfected HEK-293 cells were untreated (−) or treated (+) with Thiamet G. Cell lysates were immunoprecipitated with anti-GFP antibodies and immunoblotting was performed using antibodies against O-GlcNAc (RL-2). (**b**) Wild-type and *OGA*^+/−^ MEFs were stimulated with Wnt3a (100 ng/ml) at the indicated time points, and then subjected to immunoblotting. (**c**) *Axin2* and *Jun* mRNA levels were analyzed by qPCR after Wnt3a treatment for 6 h. (**d**) Control, OGA knockdown and Thiamet G (#SML0244; Sigma, Madison, WI, USA)-treated HEK-293 cells were stimulated with Wnt3a-conditioned media (CM), and analyzed by western blotting at the indicated time points. (**e**) The same samples were analyzed by qPCR after 6 h of stimulation. Wnt3a-conditioned media were prepared from mouse L cells (ATCC, Manassas, VA, USA) stably expressing Wnt3a. The primer sequences (human) used were: *Axin2* forward, 5′-ATGAGTAGCGCCGTGTTAGTG-3′ *Axin2* reverse, 5′-GGGCATAGGTTTGGTGGACT-3′ GAPDH forward, 5′-CCACTCCTCCACCTTTGAC-3′ and GAPDH reverse, 5′-ACCCTGTTGCTGTAGCCA-3′.

The activation of β-catenin is essential not only for tumor initiation but also for tumor progression in *Apc*^min/+^ mice.^21^ Importantly, β-catenin is directly O-GlcNAcylated, and we also observed that β-catenin is modified with O-GlcNAc (Figure 4a). The O-GlcNAcylation of β-catenin regulates its localization and transcriptional activity.^22^ OGT interacts with β-catenin to regulate cyclin D1 synthesis upon serum stimulation.^12^ Therefore, we assessed whether OGA deficiency affects Wnt/β-catenin signaling. The deletion of OGA did not affect Wnt3-mediated β-catenin accumulation (Figure 4b). In addition, there were no significant differences in *Axin2* and *Jun* mRNA levels between *OGA*^+/+^ and *OGA*^−/−^ mouse embryonic fibroblasts after stimulation with Wnt3a (Figure 4c). We also used immunoblotting and quantitative PCR to further confirm that the OGA inhibitor Thiamet G and knockdown did not affect Wnt/β-catenin signaling (Figures 4d and e). Increased O-GlcNAcylation after Thiamet G treatment might result in the upregulation OGA to compensate for the increased O-GlcNAcylation. This observation supports the results presented in Figure 3a. These results suggest that OGA heterozygosity affects tumorigenesis independently of Wnt/β-catenin signaling. In addition, intestinal tumorigenesis correlates with OGT and OGA levels, which are important for the dynamic regulation of O-GlcNAcylation.

## Conclusions

Many studies have suggested that elevated O-GlcNAcylation and/or O-GlcNAc cycling enzymes contribute to tumorigenesis. In colon cancer, the levels of O-GlcNAc and OGT are increased.^23^ However, it remains unclear whether the elevated O-GlcNAcylation caused by increased OGT activity promotes tumorigenesis *in vivo*. Here, we suggested a critical role for O-GlcNAc cycling enzymes in colorectal tumorigenesis. We observed that human colonic adenomas exhibit elevated O-GlcNAcylation, and increased levels of both OGT and OGA. Interestingly, an analysis of microarray data sets revealed that *OGA* and *OGT* expression was correlated. This supports the immunoblotting data, which showed simultaneously increased OGT and OGA in colonic adenomas. Importantly, OGT and OGA are both correlated with survival and cancer recurrence in patients with CRC. Our results demonstrate that OGA heterozygosity reduces APC-mediated colorectal tumorigenesis and enhances the survival of OGA heterozygous mice in the *Apc*^min/+^ background. Wnt target genes were downregulated in *Apc*^min/+^ *OGA*^+/−^ mice compared with *Apc*^min/+^ controls. Although β-catenin is directly O-GlcNAcylated, elevated O-GlcNAcylation did not affect Wnt/β-catenin signaling *in vitro*. Although O-GlcNAcylation is elevated in colonic adenomas, we observed reduced tumorigenesis in *OGA*^*+/*−^ mice, in which O-GlcNAcylation is constantly elevated. These results suggest that the levels of OGA and OGT are critical for colorectal tumorigenesis. These results support previous studies showing that the knockdown of OGA or OGT reduced cell growth *in vitro*.^14, 15^ This suggests that reduced levels of OGT and OGA do not properly regulate dynamic O-GlcNAcylation, which influences the functions of O-GlcNAc target proteins. Nevertheless, the mechanism by which OGA heterozygosity attenuates tumorigenesis remains unclear. However, many studies have suggested that aberrant O-GlcNAcylated proteins contribute to the disruption of cancer cell growth. Notably, many transcription factors, including c-Myc, p53, NF-κB, SP1, FoxM1 and HCF-1, are O-GlcNAcylated.^24^ O-GlcNAcylation modulates their activity or stability, which affects cancer cell growth. Phosphorylation is regulated by a variety of protein kinases and phosphatases. Although O-GlcNAcylation is similar to phosphorylation, unlike phosphorylation it is regulated by only OGT and OGA, which are encoded by single genes. Therefore, reduced levels of O-GlcNAc cycling enzymes cause various changes in the activity of entire target proteins. Furthermore, reduced levels of OGT and OGA might not normally regulate dynamic O-GlcNAcylation during the cell cycle, which is required for cell cycle control.^15, 16^ We conclude that O-GlcNAc enzymes are essential for tumor growth in CRC, and that targeting OGA may help suppress the intestinal tumorigenesis initiated by *Apc* mutation.
